# Function and Mechanism of WRKY Transcription Factors in Abiotic Stress Responses of Plants

**DOI:** 10.3390/plants9111515

**Published:** 2020-11-08

**Authors:** Weixing Li, Siyu Pang, Zhaogeng Lu, Biao Jin

**Affiliations:** College of Horticulture and Plant Protection, Yangzhou University, Yangzhou 225009, China; liwx@yzu.edu.cn (W.L.); pangsiyu_0212@163.com (S.P.); luzhaogeng@163.com (Z.L.)

**Keywords:** WRKY transcription factor, abiotic stress, gene structural characteristics, regulatory mechanism, drought, salinity, heat, cold, ultraviolet radiation

## Abstract

The WRKY gene family is a plant-specific transcription factor (TF) group, playing important roles in many different response pathways of diverse abiotic stresses (drought, saline, alkali, temperature, and ultraviolet radiation, and so forth). In recent years, many studies have explored the role and mechanism of WRKY family members from model plants to agricultural crops and other species. Abiotic stress adversely affects the growth and development of plants. Thus, a review of WRKY with stress responses is important to increase our understanding of abiotic stress responses in plants. Here, we summarize the structural characteristics and regulatory mechanism of WRKY transcription factors and their responses to abiotic stress. We also discuss current issues and future perspectives of WRKY transcription factor research.

## 1. Introduction

As a fixed-growth organism, plants are exposed to a variety of environmental conditions and may encounter many abiotic stresses, for example, drought, waterlogging, heat, cold, salinity, and Ultraviolet-B (UV-B) radiation. To adapt and counteract the effects of such abiotic stresses, plants have evolved several molecular mechanisms involving signal transduction and gene expression [1,2]. Transcription factors (TFs) are important regulators involved in the process of signal transduction and gene expression regulation under environmental stresses. TFs can be combined with *cis*-acting elements to regulate the transcriptional efficiency of target genes by inhibiting or enhancing their transcription [3,4]. Accordingly, plants may show corresponding responses to external stresses via TFs regulating target genes. Although some TF families (MYB, bZIP, AP2/EREBP, NAC) are associated with adversity [2,5], WRKY is the most extensively studied TF family in plant stress responses.

The WRKY family is a unique TF superfamily of higher plants and algae, which play important roles in many life processes, particularly in response against biotic and abiotic stress [6,7]. In 1994, the SWEET POTATO FACTOR1 (*SPF1*) gene of the WRKY family was first found in *Impoea batatas* [8]. Later, *ABF1* and *ABF2* were found in wild *Avena sativa*, and showed regulatory roles in seed germination [9]. A previous study successively cloned *WRKY1*, *WRKY2*, and *WRKY3* from *Petroselinum crispum*, named the WRKY TF, and proved for the first time that WRKY protein can regulate plant responses to pathogens [10]. With an increase in available published genomes, many members of the WRKY TF family have been identified in various species, including 104 from *Populus* [11], 37 from *Physcomitrella patens* [12], 45 from *Hordeum vulgare* [13], 55 from *Cucumis sativus* [14], 74 from *Arabidopsis thaliana* [15], 83 from *Pinus monticola* [16], 81 from *Solanum lycopersicum* [17], and 102 from *Oryza sativa* [18]. WRKY TFs exist as gene families in plants, and the number of WRKY TFs varies among species. In plants exposed to abiotic stresses (salt, drought, temperature, and so forth), WRKY family members play important roles in diverse stress responses. In addition, these TFs affect the growth and development of plants [19,20]. Therefore, WRKY TFs have attracted broad attention. Although some reviews on WRKY TFs are available, in this review we focus on the structural characteristics and regulatory mechanisms of WRKY TFs and summarize recent progress in understanding the roles of WRKY TFs during exposure to abiotic stresses such as drought, temperature, salt, and UV radiation.

## 2. Structural Characteristics of WRKY TFs

**Figure 1:**
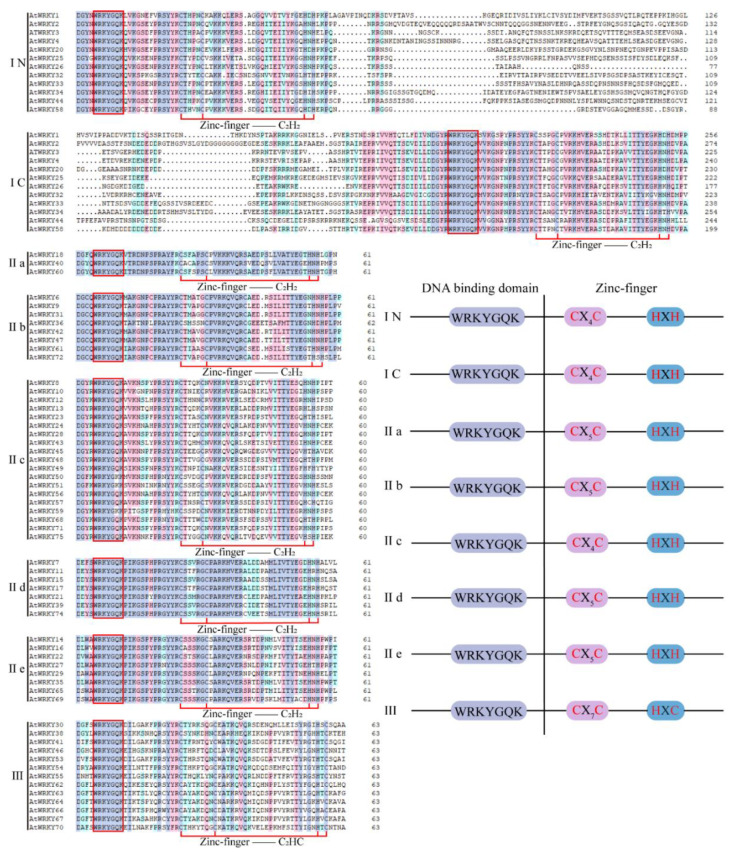
The domain of WRKY genes in *Arabidopsis thaliana*. The WRKY gene family is classified into the **I** (**I N** and **I C**), I**Ia**, **IIb**, **IIc**, **IId**, **IIe**, and **III** subfamilies. The aligned conserved domains (DNA binding and zinc-finger structures) are highlighted (left panel) and simplified (right panel).

The WRKY structure consists of two parts: the N-terminal DNA binding domain and the C-terminal zinc-finger structure [21]. The DNA binding domain sequence of WRKY is based on the heptapeptide WRKYGQK (Figure 1), but there are some differences, such as WRKYGQK, WRKYGKK, WRKYGMK, WSKYGQK, WKRYGQK, WVKYGQK, and WKKYGQK [17,22]. Zinc-finger structures mainly include C_2_H_2_ type and C_2_HC type [23], whereas some exist in the form of CX_29_HXH and CX_7_CX_24_HXC [17] (Figure 1). According to the number of WRKY domains and the structure of their zinc-finger motifs, WRKY can be divided into groups I, II, and III [23] (Figure 1). Group I usually contains two WRKY domains and one C_2_H_2_ zinc-finger structure. Those in group II and group III contain only one WRKY domain. The difference is that the zinc-finger structure in group II is C_2_H_2_ and that in group III is C_2_HC [19,21,23] (Figure 1). According to the phylogenetic relationship of the amino acid sequence of the primary structure, group II can be further divided into subgroups a–e [7,23,24]. Evolutionary analyses have shown that the WRKY of group II is not generally a single source, mainly including five categories I, IIa + IIb, IIc, IId + IIe, and III [7,24]. In addition, some WRKY proteins contain a glutamate enrichment domain, a proline enrichment domain, and a leucine zipper structure [25].

## 3. Regulatory Mechanism of WRKY TFs

WRKY family members have diverse regulatory mechanisms. Briefly, WRKY protein can be effectively combined with W-box elements to activate or inhibit the transcription of downstream target genes. Moreover, it can also bind other acting elements to form protein complexes, which enhances the activity of transcription binding [21].

WRKY TFs can effectively activate the expression of downstream genes by binding conserved W-box *cis*-acting elements in the downstream gene promoter region [21,26]. There are abundant W-box elements in the self-promoter of most WRKY TFs. Therefore, these WRKY TFs can bind with their own promoters to achieve self-regulation or cross-regulation networks by combining with other WRKY TFs [27]. For example, *CaWRKY6* of *Capsicum frutescens* can activate *CaWRKY40* and make the plant more tolerant to high temperature and humidity. *Glycine max GmWRKY27* not only inhibits the activity of downstream *GmNAC29* promoter by independent inhibition, but also cooperatively interacts with *GmMYB174* to inhibit the expression of *GmNAC29*, thereby increasing drought and salt stress resistances [28]. Moreover, chromatin immunoprecipitation (ChIP) studies have shown that when *Petroselinum crispum* is infected by pathogenic bacteria, *PcWRKY1* promoter can effectively bind to itself and the W-box of *PcWRKY3* promoter, and transcriptional activation can be achieved through self-negative feedback regulation and cross-regulation with other WRKY proteins [29]. In addition, WRKY TFs can interact with non-W-box elements. For example, *Oryza sativa OsWRKY13* can interact with PRE4 (TGCGCTT) elements [30]. *Hordeum vulgare HvWRKY46* and *Nicotiana tabacum NtWRKY12* can effectively combine with the sucrose response element SURE [31,32]. These results indicate that there are multiple binding modes between WRKY TFs and structural genes. Different binding patterns and preferences of binding sites allow for the regulation of downstream target genes, providing WRKY TFs with versatile functions in the plant transcriptional regulation network.

## 4. WRKY TF Involved in Abiotic Stress Responses

**Figure 2:**
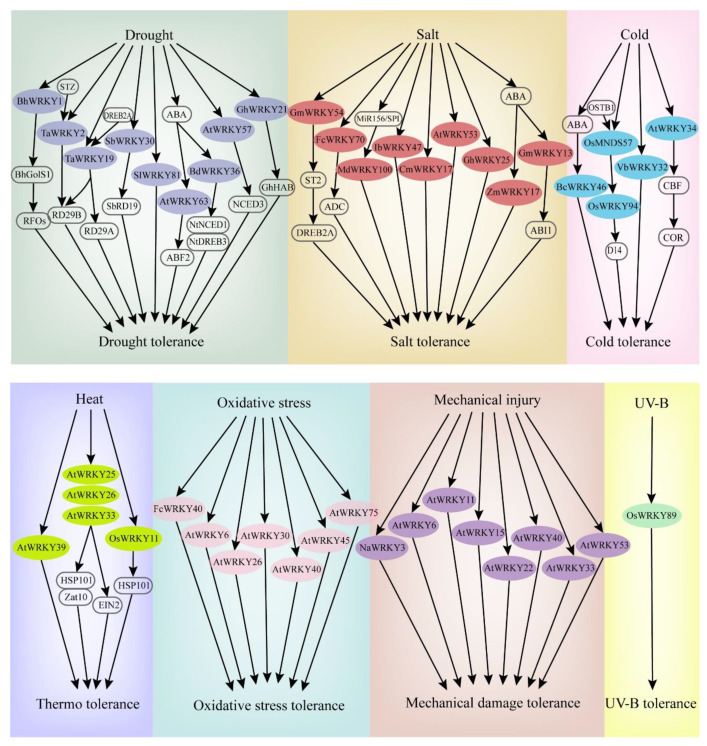
Some WRKY genes involved in the response pathways of major abiotic stresses (drought, salt, cold, heat, oxidative stress, mechanical injury, UV-B).

When plants sense stress, the corresponding signaling is activated and transferred to the cell interior. Reactive oxygen species (ROS) and Ca^2+^ ions are usually exchanged as the signal transduction in the cell. Protein kinases such as MPKs are subsequently activated to regulate the activities of related TFs. Consequently, the plant presents a stress response [31,32]. In response to abiotic stresses, some WRKY TFs can be rapidly differentially expressed, promoting signal transduction and regulating the expression of related genes [33]. The expression patterns and functional identifications of WRKYs in most studies are generally based on transcriptome analyses, real-time fluorescence quantitative PCR, gene chip analyses, and genetic transformation. Hence, WRKY genes can function effectively in most abiotic stress responses or tolerances in various plants (Table 1, Figure 2).

### 4.1. WRKY TFs and Drought Stress

Drought has a major impact on plant growth and development, resulting in a significant decrease in grain and other types of crop yield [77]. Under drought stress, drought-tolerant plants can accumulate oligosaccharides through sucrose metabolism to improve drought resistance. For example, when *Arabidopsis* is subjected to drought stress, the expression of *AtWRKY53* combined with the Qua-Quine Starch (QQS) promoter sequence is rapidly induced, hydrogen peroxide content is reduced, and the glucose metabolism pathway is significantly enhanced, thereby regulating stomatal opening and ultimately affecting drought tolerance [37]. In *Boea hygrometrica*, *BhWRKY1* effectively regulates the expression of the *BhGolS1* gene, and the overexpression of *BhGolS1* and *BhWRKY1* induces the accumulation of raffinose family oligosaccharides (RFOs) in transgenic *Nicotiana tabacum*, thus improving the ability of seedlings to resist drought [60].

WRKY protein can directly regulate the expression of drought-resistant genes. For example, in sorghum, *SbWRKY30* regulates the drought stress response gene *SbRD19* by binding with W-box elements of the *SbRD19* promoter, and protects plant cells from the damage of reactive oxygen species by improving ROS scavenging capability, enhancing drought tolerance [66]. *TaWRKY2* of wheat can bind to *STZ* and downstream drought-resistant gene *RD29B* promoter, with a positive regulatory effect on the expression of *RD29B* [58]. *DREB2A* regulates the expression of dehydration stress-related genes [78], while *TaWRKY19* can bind to *DREB2A* promoter, ultimately activating the expression of *RD29A*, *RD29B*, and *Cor6*.*6* in transgenic *Arabidopsis* plants [58]. Similarly, *Arabidopsis AtWRKY57* positively regulates the expression of *RD29A* and *NCED3* genes by binding their W-box elements in the promoter regions [39]. In addition, WRKY protein can act on other TFs to play regulatory roles in drought tolerance. For example, *TcWRKY53* of *Thlaspi arvense* significantly inhibits the expression of *NtERF5* and *NterEBp-1* of the AP2/ERF TF family, thus improving plant resistance to drought stress [63].

WRKY TFs also regulate plant tolerance through abscisic acid (ABA) and ROS-related signaling pathways. During drought stress, higher ABA levels were accumulated in plants, and leaf stomata were closed to reduce transpiration rate, thus regulating water balance in plants. ABA accumulation in cells, integrated with a variety of stress signals, regulates the expression of downstream genes, consequently sensing and responding to the adverse environment [40]. *Arabidopsis AtWRKY63* has a specific effect on ABA-mediated stomatal closure and other signal transduction pathways, thus affecting the drought response [40]. *GhWRKY21* regulates ABA-mediated cotton drought tolerance by promoting the expression of *GhHAB* [43]. Overexpression of *BdWRKY36* in tobacco reduces the accumulation of ROS, activated *NtLEA5*, *NtNCED1*, and *NtDREB3* in the ABA biosynthetic pathway, and significantly enhances the drought resistance of plants [48]. In *Solanum lycopersicum*, *SlWRKY81* increases the drought tolerance of plants by inhibiting the accumulation of H_2_O_2_, playing a negative regulation role of stomatal closure [72].

### 4.2. WRKY TFs and Salt Stress

Salt stress is an important abiotic stress affecting crop productivity, particularly in arid and semiarid regions. WRKY TFs play essential roles in regulating the response to salt stress. To date, a total of 47 WRKY genes have been found to be expressed under salt stress in the wheat genome [79]. *STZ* is a protein related to ZPT2, which acts as a transcriptional inhibitor to downregulate the deactivation of other transcription factors. *GmWRKY54* of *Glycine max* inhibits *STZ* expression and responds to salt stress by positively regulating the DREB2A-mediated pathway [55]. *FcWRKY70* promotes the upregulation of arginine decarboxylase (ADC) expression, which is heterologously expressed in tobacco, and the content of lemon putrescine is significantly increased, thus enhancing the salt tolerance of plants [49]. The *IbWRKY47* gene positively regulates stress resistance-related genes and significantly improves the salt tolerance of *Ipomoea batatas* [68]. MiR156/SPL modulates salt tolerance by upregulation of *Malus domestica* salt tolerance gene *MdWRKY100* [71]. In *Sorghum bicolor*, *SbWRKY50* could directly bind to the upstream promoter of *SOS1* and *HKT1* and participate in plant salt response by controlling ion homeostasis [67]. In addition, some *WRKY* genes function as negative regulation factors involved in salt stress resistance. *Arabidopsis* RPD3-like histone deacetylase HDA9 inhibits salt stress tolerance by regulating the DNA binding and transcriptional activity of *WRKY53* [38]. *Chrysanthemum CmWRKY17* overexpressed in *Arabidopsis* allows the plants to be more sensitive to salt stress. The expression level of stress resistance-related genes in transgenic *Arabidopsis* is lower than that in wild-type plants, indicating that *CmWRKY17* may be involved in negatively regulating the salt stress response in *Chrysanthemum* [80]. The expression of *GhWRKY68* is strongly induced in upland cotton and decreases salt tolerance [45]. In contrast, a high expression level of *GhWRKY25* enhances the salt tolerance of upland cotton, while transgenic tobacco shows a relatively weaker tolerance to drought stress [44], indicating that the regulatory effects of different WRKY TFs involved in drought response are different.

Plants can also respond to saline–alkali stress through ABA, H_2_O_2_, and other signal pathways. In *Glycine max*, the negative regulatory factor *ABI1* in the ABA pathway may be the downstream target gene of *GmWRKY13*. Genetic transformation experiments in *Arabidopsis* have shown that overexpression of *GmWRKY13* significantly increases the expression of *ABI1*, but plants show a low tolerance to salt stress [55]. Overexpression of *ZmWRKY17* has an inhibitory effect on the sensitivity of exogenous ABA treatment, resulting in a relatively lower tolerance to high levels of salinity [57]. Under salt-induced H_2_O_2_ and cytosolic Ca^2+^ stimulation, the activity of antioxidant enzymes increases, thus improving the tolerance to high-salinity environments [81]. ABA-induced WRKY gene expression is largely related to salt stress. Exogenous application of ABA and NaCl also induce *AtWRKY25* and *AtWRKY33* in *Arabidopsis* [33], *OsWRKY72* in rice [51], *GbWRKY1* in *Verbena bonariensis* [73], and *VpWRKY1/2* [61] and *VpWRKY3* [62] in grape.

### 4.3. WRKY TFs and Temperature Stress

Both low- and high-temperature stress can reduce crop yield and quality in plants. WRKY TFs play a role in the stress response through different signal transduction pathways. For example, in *Verbena bonariensis*, *VbWRKY32* as a positive regulator, upregulates the transcriptional level of cold response genes, which increases the antioxidant activity, maintains membrane stability, and enhances osmotic regulation ability, thereby improving the survival ability under cold stress [74]. The *BcWRKY46* gene of *Brassica campestris* is strongly induced by low temperature and ABA, activating related genes in the ABA signaling pathway to improve the low-temperature tolerance of plants [59]. *CBF* TFs regulate the expression of *COR*, and the overexpressed transgenic lines of *CBF1*, *CBF2*, and *CBF3* show stronger cold resistance [82]. *AtWRKY34* has a negative regulatory effect on the CBF-mediated cold response pathway; it is specifically expressed in mature pollen grains after exposure to low temperatures, resulting in resistance to low temperatures [35]. In addition, plants respond to temperature changes by coordinating organ development in an adverse environment. At low temperatures, rice MADS-Box TF *OsMADS57* and its interacting protein *OsTB1* synergistically activate the transcriptional regulation of *OsWRKY94*, preventing tillering by inhibiting transcription of the organ development gene *D14* [83].

Due to global climate change, high-temperature stress has attracted significant attention. There is evidence that, to a certain extent, high temperatures will lead to biochemical changes in plants [84]. Thermal stimulation can activate Ca^2+^ channels to maintain a higher intracellular Ca^2+^ concentration, thereby activating calmodulin protein expression and inducing thermal-shock protein transcriptional expression [85]. In *Arabidopsis*, *AtWRKY54* significantly responds to heat shock whereas basic leucine zipper factors (bZIPs) respond to prolonged warming [41]. Overexpression of *AtWRKY39* can make plants more heat-sensitive. *AtWRKY39* is highly homologous to *AtWRKY7*, and both of them can effectively bind calmodulin in plants, indicating a similar function [36]. In addition, *AtWRKY25*, *AtWRKY26*, and *AtWRKY33* can improve tolerance to high-temperature stress in transgenic *Arabidopsis* by regulating the *Hsp101* and *Zat10* genes [34]. Plants subjected to heat stress can also activate the oxidative stress response through ethylene [86]. Under high-temperature stress, the expressions of *AtWRKY25*, *AtWRKY26*, and *AtWRKY33* in *Arabidopsis* are induced by ethylene, the feedback factor *EIN2* is transcriptionally regulated, and the effective activation of ethylene signal transduction contribute to relatively stronger heat resistance. In *Oryza sativa*, *HSP101* promoter can activate the expression of the *OsWRKY11* gene. Under heat treatment, the leaves wilted more slowly and the green part of the plant was less damaged, which makes it more heat-resistant [50]. In addition, some noncoding RNAs, such as miR396, play a role in the response to heat stress by regulating its target *WRKY6* [87].

### 4.4. WRKY TFs and Other Abiotic Stresses

**Figure 3:**
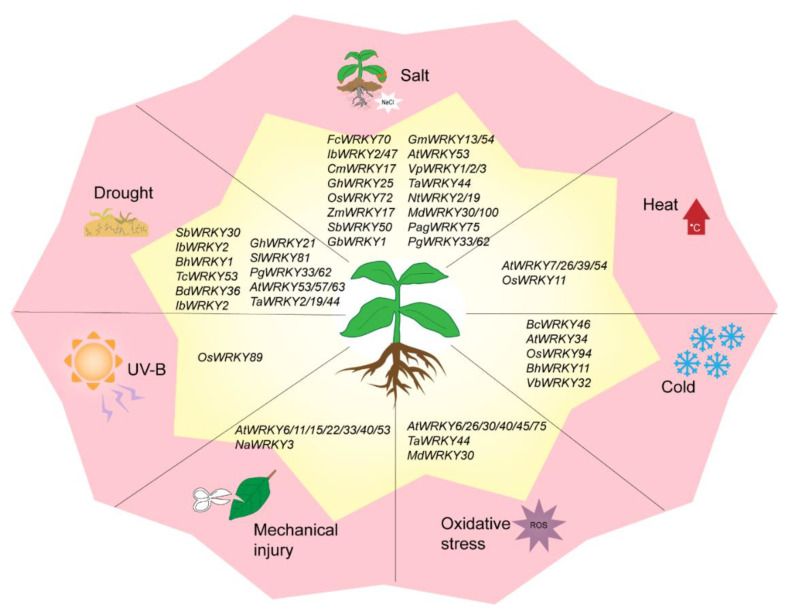
WRKY transcription factors in response to abiotic stresses.

WRKY TFs are also involved in oxidative stress, mechanical damage, UV radiation, and other abiotic stresses (Figure 3). *FcWRKY40* overexpression can significantly enhance the resistance of transgenic tobacco to oxidative stress [88]. When *Arabidopsis* is treated with ROS, the expressions of *AtWRKY30*, *AtWRKY40*, *AtWRKY75*, *AtWRKY6*, *AtWRKY26*, and *AtWRKY45* are significantly upregulated [89]. After mechanical injury, the expression levels of *AtWRKY11*, *AtWRKY15*, *AtWRKY22*, *AtWRKY33*, *AtWRKY40*, *AtWRKY53* [90] and *AtWRKY6* [64] are upregulated. Similarly, *NaWRKY3* is strongly expressed in tobacco. By contrast, the sensitivity of transgenic plants is increased when *NaWRKY3* is knocked out [64]. In two previous studies, UV-B radiation treatment induced three WRKY genes in *Arabidopsis* and the *OsWRKY89* gene in rice, resulting in a thick waxy substance on the leaf surface and improved tolerance to heat [54,91].

In addition, a single WRKY TF can play multiple roles in different stress responses via various signal pathways and regulatory networks. For example, *TaWRKY44* expression in tobacco can improve resistance to drought, salt stress, and osmotic stress [92], while *PgWRKY62* and *PgWRKY33* in *Pennisetum glaucum* respond to salt and drought simultaneously [75]. *BhWRKY1* protein in *Boea hygrometrica* binds to the promoter of *BhGolS1* and is associated with both low-temperature resistance and drought tolerance [60]. *IbWRKY2* can interact with *IbVQ4*, and drought and salt treatment can induce the expression of *IbVQ4*, thus improving the tolerance of plants to drought and salt stress [69]. *MdWRKY30* overexpression enhances tolerance to salt and osmotic stress in transgenic apple callus through transcriptional regulation of stress-related genes [70]. *PagWRKY75* negatively regulates the tolerance of 84 K poplar (*Populus alba* × *P. glandulosa*) to salt and osmotic stress by reducing the scavenging capacity of ROS and the accumulation of proline, thus actively regulates the rate of leaf water loss [76].

## 5. Conclusions and Perspectives

As one of the largest TF families, WRKY plays an important and indispensable role in normal life activities of plants. Over the years, it has been shown that WRKY TFs not only participate in plant growth and development, but also show complex regulatory mechanisms and networks involved in external abiotic stresses. A large number of WRKYs have been functionally characterized in model plants, providing abundant functional references for other plants. Given that crops usually face various stresses and WRKYs play important roles in stress responses, further in-depth studies on WRKY genes in more crops are required. As increasing plant genomes have been sequenced, particularly of economically important crops, the genome-wide identification of WRKY genes will facilitate screening for stress resistance-related functional genes in plants. Moreover, previous studies of WRKY gene functions were largely dependent on transcriptomics and functional predictions, whereas more applications of genetic verification combined with new technologies are accelerating the research progress of WRKY’s novel functions. In addition, characterization of the downstream genes regulated by WRKY TFs or WRKY TF self-regulation will help clarify the regulatory network of abiotic stress responses. Furthermore, noncoding RNAs and epigenetic modifications involved in the regulation of WRKY TFs should be explored in future studies. Ultimately, using WRKY TFs to screen for stress-resistant plant cultivars and improve plant stress resistance will significantly benefit agricultural crop yield and quality in the context of aggravated climate change.

## Figures and Tables

**Table 1:** WRKY transcription factors (TFs) involved in abiotic stress responses in plants.

No.	Gene	Species	Induced by Factors	Function	References
1	*AtWRKY25/26*	*Arabidopsis*	Heat	Tolerance to heat	[34]
2	*AtWRKY33*	*Arabidopsis*	NaCl, mannitol, H_2_O_2_	Tolerance to heat and NaCl, negative regulator in oxidative stress and abscisic acid (ABA)	[33]
3	*AtWRKY34*	*Arabidopsis*	Cold	Negative regulator in cold stress	[35]
4	*AtWRKY39*	*Arabidopsis*	Heat	Tolerance to heat	[36]
5	*AtWRKY53*	*Arabidopsis*	Drought, salt	Reduced drought resistance and H_2_O_2_, sensitive to salt	[37,38]
6	*AtWRKY57*	*Arabidopsis*	Drought	Tolerance to drought	[39]
7	*AtWRKY63*	*Arabidopsis*	ABA	Tolerance to drought, regulated ABA signaling	[40]
8	*AtWRKY54*	*Arabidopsis*	Heat	Response to heat stress	[41]
9	*POWRKY13*	*Populus tomentosa*	Heat	Response to heat stress	[42]
10	*GhWRKY21*	*Gossypium hirsutum*	Drought	Tolerance to drought	[43]
11	*GhWRKY25*	*Gossypium hirsutum*	Drought	Tolerance to salt, reduced drought resistance	[44]
12	*GhWRKY68*	*Gossypium hirsutum*	Salt, drought	Reduced salt tolerance and drought resistance, positive regulator in ABA signaling	[45]
13	*VvWRKY24*	*Vitis vinifera*	Cold	Upregulated expression at all stages of hypothermia	[46]
14	*CaWRKY40*	*Capsicum annuum*	Heat	Tolerance to heat	[47]
15	*BdWRKY36*	*Brachypodium distachyon*	Drought	Tolerance to drought	[48]
16	*FcWRKY70*	*Fortunella crassifolia*	Salt	Tolerance to salt	[49]
17	*OsWRKY11*	*Oryza sativa*	Heat, drought	Tolerance to drought and heat	[50]
18	*OsWRKY72*	*Oryza sativa*	Drought, NaCl, ABA	Sensitive to salt, drought, sucrose, and ABA	[51]
19	*OsWRKY74*	*Oryza sativa*	Pi deprivation, cold	Tolerance to cold and Pi deprivation	[52]
20	*OsWRKY76*	*Oryza sativa*	Cold	Tolerance to cold	[53]
21	*OsWRKY89*	*Oryza sativa*	ABA, UV-B	Tolerance to UV	[54]
22	*GmWRKY13*	Soybean	Salt, drought	Sensitive to salt and mannitol, negative regulator in ABA signaling	[55]
23	*GmWRKY17*	Soybean	Salt	Reduced salt tolerance	[56]
24	*GmWRKY54*	Soybean	Salt, drought	Tolerance to salt and drought	[55]
25	*GmWRKY21*	*Glycine max*	NaCl, drought, cold	Tolerance to cold	[55]
26	*ZmWRKY17*	*Zea mays*	ABA, salt	Reduced salt tolerance	[57]
27	*TaWRKY2/19*	*Triticum aestivum*	NaCl, drought, ABA	Tolerance to salt and drought	[58]
28	*BcWRKY46*	*Brassica campestris*	NaCl, drought, cold	Tolerance to salt and drought	[59]
29	*BhWRKY1*	*Boea hygrometrica*	Dehydration, ABA	Tolerance to drought	[60]
30	*VpWRKY1*	*Vitis pseudoreticulata*	NaCl, ABA	Tolerance to salt	[61]
31	*VpWRKY2*	*Vitis pseudoreticulata*	Cold, NaCl, ABA	Tolerance to salt and cold	[61]
32	*VpWRKY3*	*Vitis pseudoreticulata*	Drought, ABA, salicylic acid (SA)	Tolerance to salt	[62]
33	*TcWRKY53*	*Thlaspi caerulescens*	Cold, PEG, NaCl	Negative regulator in osmotic stress	[63]
34	*NaWRKY3*	*Nicotiana attenuate*	Mechanical damage	Sensitive to mechanical damage	[64]
35	*JrWRKY2/7*	*Juglans regia*	Drought, cold	Tolerance to drought and cold	[65]
36	*SbWRKY30*	*Sorghum bicolor*	Salt, drought	Tolerance to salt and drought	[66]
37	*SbWRKY50*	*Sorghum bicolor*	Salt	Tolerance to salt	[67]
38	*IbWRKY47*	*Ipomoea batatas*	Salt	Tolerance to salt	[68]
39	*IbWRKY2*	*Ipomoea batatas*	Salt, drought	Tolerance to salt and drought	[69]
40	*MdWRKY30*	*Malus domestica*	Salt, osmotic stress	Tolerance to salt and osmotic stress	[70]
41	*MdWRKY100*	*Malus domestica*	Salt	Sensitive to salt	[71]
42	*SlWRKY81*	*Solanum lycopersicum*	Drought	Reduced drought tolerance	[72]
43	*GbWRKY1*	*Gossypium barbadense*	Salt	Tolerance to salt	[73]
44	*VbWRKY32*	*Verbena bonariensis*	Cold	Tolerance to cold	[74]
45	*PgWRKY33/62*	*Pennisetum glaucum*	Salt, drought	Tolerance to salt and drought	[75]
46	*PagWRKY75*	*Populus alba*	Drought	Negative regulator in salt and osmotic stress	[76]
